# A population pharmacokinetic model for posaconazole intravenous solution and oral powder for suspension formulations in pediatric patients with neutropenia

**DOI:** 10.1128/aac.01197-23

**Published:** 2024-02-20

**Authors:** Gregory Winchell, Rik de Greef, Aziz Ouerdani, Floris Fauchet, Rebecca E. Wrishko, Eric Mangin, Christopher Bruno, Hetty Waskin

**Affiliations:** 1 Certara USA, Inc., Princeton, New Jersey, USA; 2 Certara France, Paris, France; 3 Merck & Co., Inc., Rahway, New Jersey, USA; The University of Iowa, Iowa City, Iowa, USA

**Keywords:** antifungal agents, invasive fungal disease, pediatrics, pharmacokinetics, posaconazole

## Abstract

The objective of this study was to support posaconazole dose regimens in pediatric patients aged ≥2 years, using a population pharmacokinetic (PK) approach with data from a phase 1b study (NCT02452034). A one-compartment model with first-order absorption was fit to pharmacokinetic data from 144 participants aged 2 to 17 years, who were administered posaconazole as intravenous (IV) and powder for oral suspension (PFS) formulations, or IV only, at dosing regimens of 3.5, 4.5, and 6 mg/kg. The influence of demographic and clinical factors on pharmacokinetic parameters was evaluated using a stepwise forward inclusion/backward exclusion procedure. The final model simulated posaconazole exposure in patients aged 2 to <7 and 7 to 17 years at dosing regimens of 4.5, 6, and 7.5 mg/kg. Plasma concentration data following IV and PFS administration were well-described by a one-compartment model with first-order absorption and estimated bioavailability, where clearance and volume were subject to allometric scaling by body weight. The 6-mg/kg dosing regimen achieved the pharmacokinetic target (90% of the pediatric population having an average steady-state plasma concentration of ≥500 and <2,000 ng/mL) for both age groups, regardless of whether patients received IV and PFS or IV only. In a virtual adolescent population (body weight >40 kg), the 300 mg/day posaconazole tablet was also predicted to achieve the pharmacokinetic target and remain within a safe range of exposure. These data informed a weight-based nomogram for PFS dosing to maximize the number of pediatric patients achieving the pharmacokinetic target across weight bands, while also maintaining a favorable benefit/risk profile.

## INTRODUCTION

Invasive fungal disease (IFD) is a leading cause of infectious disease morbidity and mortality in immunocompromised patients, especially in patients with severe and persistent neutropenia and T-cell dysfunction (1–3). As in adults, children at risk for IFD include allogenic hematopoietic stem cell transplant (HSCT) recipients and patients with acute leukemia, myelodysplasia, severe aplastic anemia, or advanced-stage non-Hodgkin lymphoma undergoing chemotherapy (3–6).

Posaconazole is a triazole antifungal approved in the United States and Europe for the prophylaxis of IFD resulting from *Aspergillus, Candida,* and other fungal species in severely immunocompromised patients; it is also effective in the treatment of IFDs refractory to other antifungal treatments (7–9). Like other azole antifungals, posaconazole inhibits the fungal cytochrome (CYP)-dependent enzyme, lanosterol 14 α-demethylase, thereby blocking the biosynthesis of ergosterol and impairing cell membrane stability (10). It is available in three formulations, namely, an intravenous (IV) solution, an oral suspension, and an oral tablet with improved bioavailability (8, 9). The recommended dosing regimen in adults is a loading dose of 300 mg twice daily (BID) on day 1, followed by 300 mg once daily (QD) as a maintenance dose. In a quartile analysis conducted by the US Food and Drug Administration, an average steady-state plasma concentration (C_avg_) of ≥500 ng/mL was shown to be associated with an increase in clinical response in adults (11).

The goal of the posaconazole pediatric program was to utilize the established exposure–response relationship of posaconazole in adults to identify a pediatric dosing strategy that would achieve a pharmacokinetic (PK) target of a mean C_avg_ of 1,200 ng/mL, in which approximately 90% of pediatric patients would have a C_avg_ of ≥500 and <2,500 ng/mL. Furthermore, an upper exposure limit for toxicity has not been established for posaconazole. The results of a previous study on adult patients at high risk of IFD showed no correlation between high exposure levels (predicted C_avg_ of 2,304 to 9,523 ng/mL) and the occurrence of treatment-related adverse events for a posaconazole tablet formulation (12). In a previous posaconazole pediatric study, pediatric patients aged 2 to 17 years with neutropenia were treated with an oral suspension of 12 or 18 mg/kg per day (divided BID); however, population PK analyses determined that the oral suspension of posaconazole would likely not reach the defined PK targets (13), and a powder for oral suspension (PFS) formulation was developed with improved bioavailability similar to that of the marketed oral tablet (8). The PFS is a pH-controlled delayed-release formulation containing amorphous posaconazole in the form of a solid dispersion powder; it provides convenient oral administration and dosing flexibility in pediatric patients aged 2 to 17 years (14).

In a phase 1b open-label, sequential dose-escalation trial in pediatric patients aged 2 to 17 years with documented or expected neutropenia (P097; NCT02452034), the PK profiles of PFS and IV formulations were evaluated at doses of 3.5, 4.5, and 6 mg/kg (14). Data from this study suggested that both the 4.5 and 6 mg/kg dosing schedules achieved a C_avg_ of ≥500 ng/mL in approximately 90% of participants. The IV and PFS posaconazole formulations were well-tolerated in pediatric patients, and the safety profiles were comparable to those reported in adults (14). Similar to the weight-based dosing regimens, PK profiles were also simulated with the results stratified by body weight to explore fixed dosing by the weight band for simplification of administration instructions (8). Here, we report on the results of a population PK analysis of the P097 study to further support dosing recommendations in pediatric patients aged 2 to 17 years.

## RESULTS

### Participant characteristics

The analysis data set included 1,236 PK observations from 114 pediatric participants aged 2 to 17 years (Table 1). About 59% of the population was male, and a majority of the participants were White (83%) and not Hispanic (87%). Participants were administered posaconazole IV (*n* = 54) or IV and PFS (*n* = 60) at doses of 3.5 (*n* = 35), 4.5 (*n* = 31), or 6 mg/kg (*n* = 48) (Table S1). A total of 80 samples were excluded from the analysis, including 33 samples from 2 participants with incomplete oral PFS dose intakes. Samples for which the sampling time was not available (31 samples) or which had the same sampling time (13 samples) were also excluded.

**TABLE 1:** PK population baseline characteristics^*a*^

Characteristic	2 to <7 years (*n* = 48)	7 to 17 years (*n* = 66)	All (*N* = 114)
Median age (range), years	3 (2–6)	13 (7–17)	8 (2–17)
Median weight (range), kg	16 (10.2–41.7)	45.4 (18.2–102)	28.6 (10.2–102)
Median BMI (range), kg/m^2^	15.5 (12.8–30.7)^*b*^	18 (12.6–33.7)^*b*^	16.5 (12.6–33.7)^*c*^
Median BSA (range), m^2^	0.662 (0.475–1.1)^*b*^	1.39 (0.793–2.26)^*b*^	1.02 (0.475–2.26)^*c*^
Median eGFR (range), ml/min/1.73 m^2^	154 (55.3–399)^*b*^	138 (12.2–314)^*b*^	146 (12.2–314)^*c*^

^*a*^ BMI, body mass index; BSA, body surface area; eGFR, estimated glomerular filtration rate.

^*b*^ Data missing for one patient.

^*c*^ Data missing for two patients overall.

### Final model

The final structural model was described using a one-compartment model, with first-order absorption, interindividual variability (IIV) on clearance (CL), central volume of distribution (V_c_) and bioavailability, correlation between CL and V_c_, estimated allometric exponent on CL and V_c_, estimated bioavailability using a logit function (in order to constrain its value between 0 and 1), and an additive error model in the logarithmic scale. Parameter estimates for the final structural model are shown in Table 2. All model parameters were well-estimated, with a percentage of relative standard error (RSE) of <20% for fixed-effect parameters and <30% for random-effect parameters. The estimate of allometric exponents for body weight on CL (0.624) and V_c_ (0.971) approximated theoretical values (0.75 and 1, respectively). Estimates of shrinkage suggested a robust estimate of the individual values for CL (shrinkage 5%), whereas the estimates for both V_c_ and bioavailability were subject to shrinkage (34% and 42%, respectively) (File S1).

**TABLE 2:** Parameter estimates for the final PK model^*a*^

Parameters	Estimate	RSE (%)	Shrinkage (%)
Fixed effects			
CL (L/h)	4.71	3.86	
V_c_ (L)	112	5.18	
KA (h^−1^)	0.212	17.9	
F1	0.826	5.58	
α for CL	0.624	9.86	
α for V_c_	0.971	7.86	
Random effects			
IIV (CL)^*b*^	37.1	8.15	5
IIV (V_c_)^*b*^	27.7	24.75	34
IIV (F1)^*c*^	2.02	18.9	42
Residual error, SD	0.331	4.71	8

^*a*^ α, allometric scaling exponent; CL, clearance; F1, relative bioavailability; IIV, interindividual variability; KA, absorption rate constant; RSE, relative standard error; SD, standard deviation; Vc, central volume of distribution.

^*b*^ Expressed as a CV%, calculated by the square root of Ω, multiplied by 100.

^*c*^ The variability in F1 is shown as a standard deviation in the logit domain. This corresponds to 95% of patients having F1 comprising between 8% and 99%.

**TABLE 3:** Proportions of participants achieving specific ranges of simulated C_avg_ and C_min_ across dose regimens of 4.5, 6, or 7.5 mg/kg QD by the age group^*a*^

Dose	Parameter	Formulation	2 to <7 years of age			7 to 17 years of age		
			Concentration ranges (ng/mL)					
			<500	≥500 to <2,500	≥2,500	<500	≥500 to <2,500	≥2,500
4.5 mg/kg	C_avg_, %	IV	2.8	95.6	1.6	0.8	93.4	5.8
		PFS	17.2	82.1	0.7	7.6	89.9	2.5
	C_min_, %	IV	45.3	54.6	0.1	20.7	78.2	1.1
		PFS	48.2	51.5	0.3	23.9	75.2	0.9
6 mg/kg	C_avg_, %	IV	0.2	94.4	5.4	0.1	82.2	17.7
		PFS	6.8	91.2	2.0	3.1	89.1	7.8
	C_min_, %	IV	30.2	69.1	0.7	9.7	85.9	4.4
		PFS	30.9	67.9	1.2	14.1	83.2	2.7
7.5 mg/kg	C_avg_, %	IV	0	83.2	16.8	0	70.2	29.8
		PFS	2.9	89.3	7.8	1.9	82.1	16.0
	C_min_, %	IV	20.8	77.7	1.5	6.4	85.4	8.2
		PFS	20.0	78.1	1.9	9.7	84.2	6.1

^*a*^ C_avg_, average plasma concentration during the dosing interval; C_min_, trough plasma concentration; IV, intravenous; PFS, powder for oral suspension; QD, once daily.

No significant relationships were found between covariates assessed in the stepwise analysis (age, weight, estimated glomerular filtration rate [eGFR], sex, and ethnicity) and CL, V_c_, or bioavailability (File S2 for age and weight; the results were similar for eGFR, sex, and ethnicity [data not shown]); therefore, the base structural model was considered the final model.

### Effect of food

**Fig 1:**
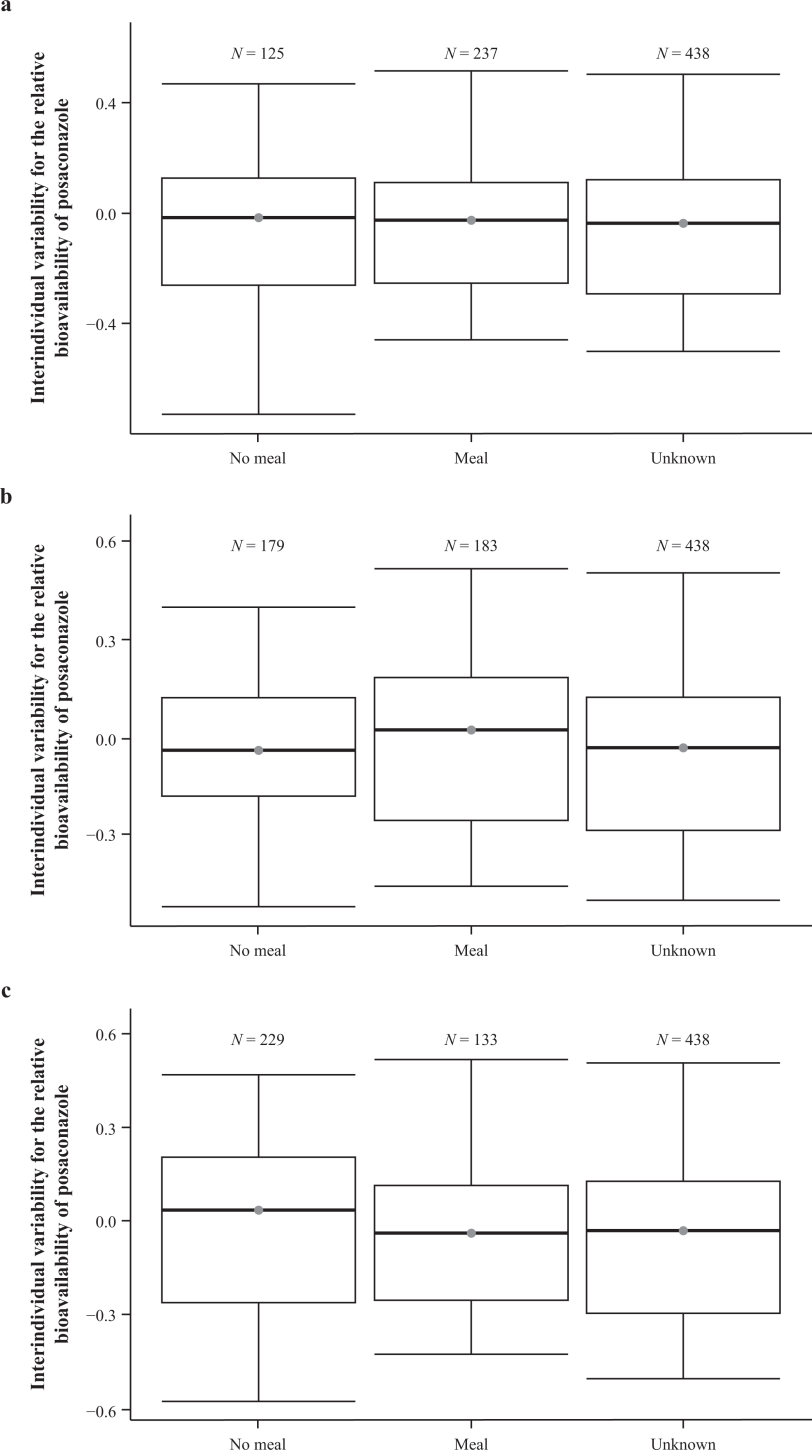
Correlation of interindividual variability with the relative bioavailability of the posaconazole PFS formulation vs two-category food effect covariates. Graphical assessments of the IIV of the posaconazole PFS formulation for (a) the overall food effect parameter, (b) food intake within 2 hours before dosing, and (c) food intake within 1 hour post-dose. N represents the number of observations with certain associated food information. The line in the box represents median, boxes extend to the 25th and 75th percentiles, and whiskers extend to the 5th and 95th percentiles. F1, relative bioavailability; IIV, interindividual variability; PFS, powder for oral suspension; POS, posaconazole.

The effect of food conditions on the bioavailability of the posaconazole PFS formulation was assessed after completion of the stepwise covariate analysis. The food effect was captured through different covariates and included the effect of no meal vs that of a meal and the effect of no meal vs that of a light meal, medium meal, and heavy meal, within 2 hours before dosing and up to 1-hour post-dose. None of the food covariates or categorizations showed a significant effect on bioavailability. Graphical assessments for the effect of no meal vs that of a meal did not indicate any clear trends to suggest that food had an impact on the bioavailability of posaconazole PFS (Fig. 1), and its inclusion did not result in a statistically significant improvement in the fit of the population PK model. Therefore, the food effect was not included in the final model.

### Model validation and simulations

**Fig 2:**
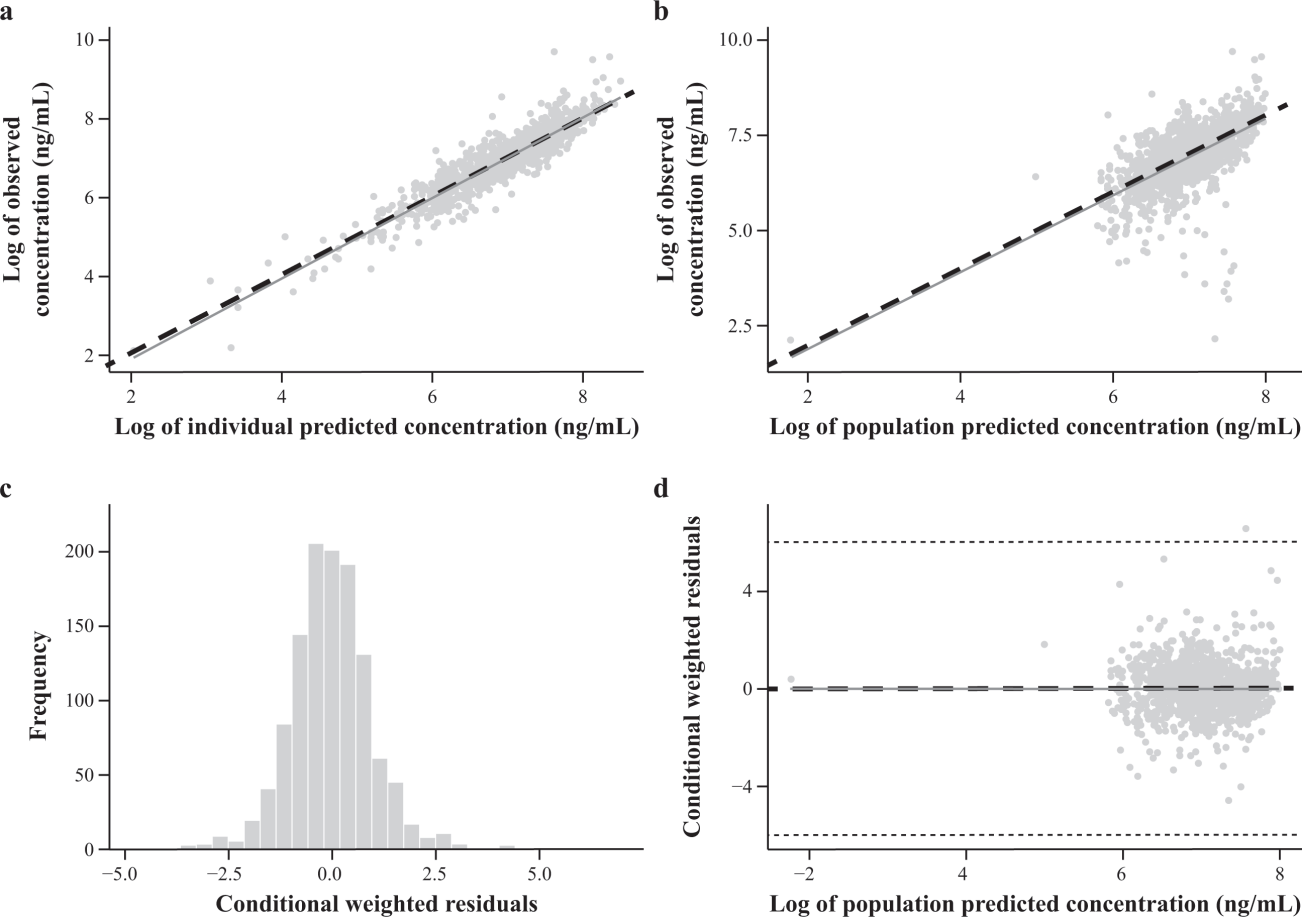
Goodness-of-fit analysis for the final structural pharmacokinetic model. Goodness-of-fit plots for (a) log of observed posaconazole concentrations (ng/mL) vs the log of individual predicted posaconazole concentrations (ng/mL), (b) log of observed posaconazole concentrations (ng/mL) vs the log of population predicted posaconazole concentrations (ng/mL), (c) frequency of individual conditional weighted residuals, and (d) conditional weight residuals vs the log of population-predicted posaconazole concentrations (ng/mL). Excludes outlier observations. In (a), (b), and (d), dots represent individual data and solid gray lines represent trend lines. In (a) and (b), the black dashed lines are lines of identity. In (d), the black dashed line represents a reference (zero) line.

Reliability of the final model was confirmed with diagnostic plots, visual predictive checks, and bootstrap analysis. The predictive performance of the population PK model was confirmed by the agreement between model-predicted and observed concentration–time values across all cohorts with no obvious bias (Fig. 2).

The robustness and fidelity of the final PK model were confirmed through the comparison of C_avg_ and trough plasma concentration (C_min_), calculated using the model-based *post hoc* parameter estimates and the corresponding noncompartmental analysis-derived PK parameters based on intensive PK sampling obtained at a steady state after approximately 10 days of IV or oral PFS administration in PN097. Overall, there was good concordance between model-predicted *post hoc* estimates of individual C_avg_ and C_min_ parameters and those calculated by noncompartmental analysis for both IV and oral PFS administration, although in the 6 mg/kg per day cohort, the model tended to overpredict C_min_ for the PFS administration (File S3).

Simulation-based visual predictive checks showed that the model accurately tracked the central tendency of the observed data (i.e., the observed data are symmetrically distributed around the median predictions, with a majority lying within the 90% prediction interval of the model predictions), providing further confirmation that the final model adequately described the observed data when stratified by age and route of administration (File S4). Taken together, these results support the fidelity of the model over the entire age range (2 to 17 years) following both IV and oral PFS administration and support the use of the model in confirming the appropriateness of the 6 mg/kg per day dose and exploring alternative dosing regimens using simulation approaches.

**Fig 3:**
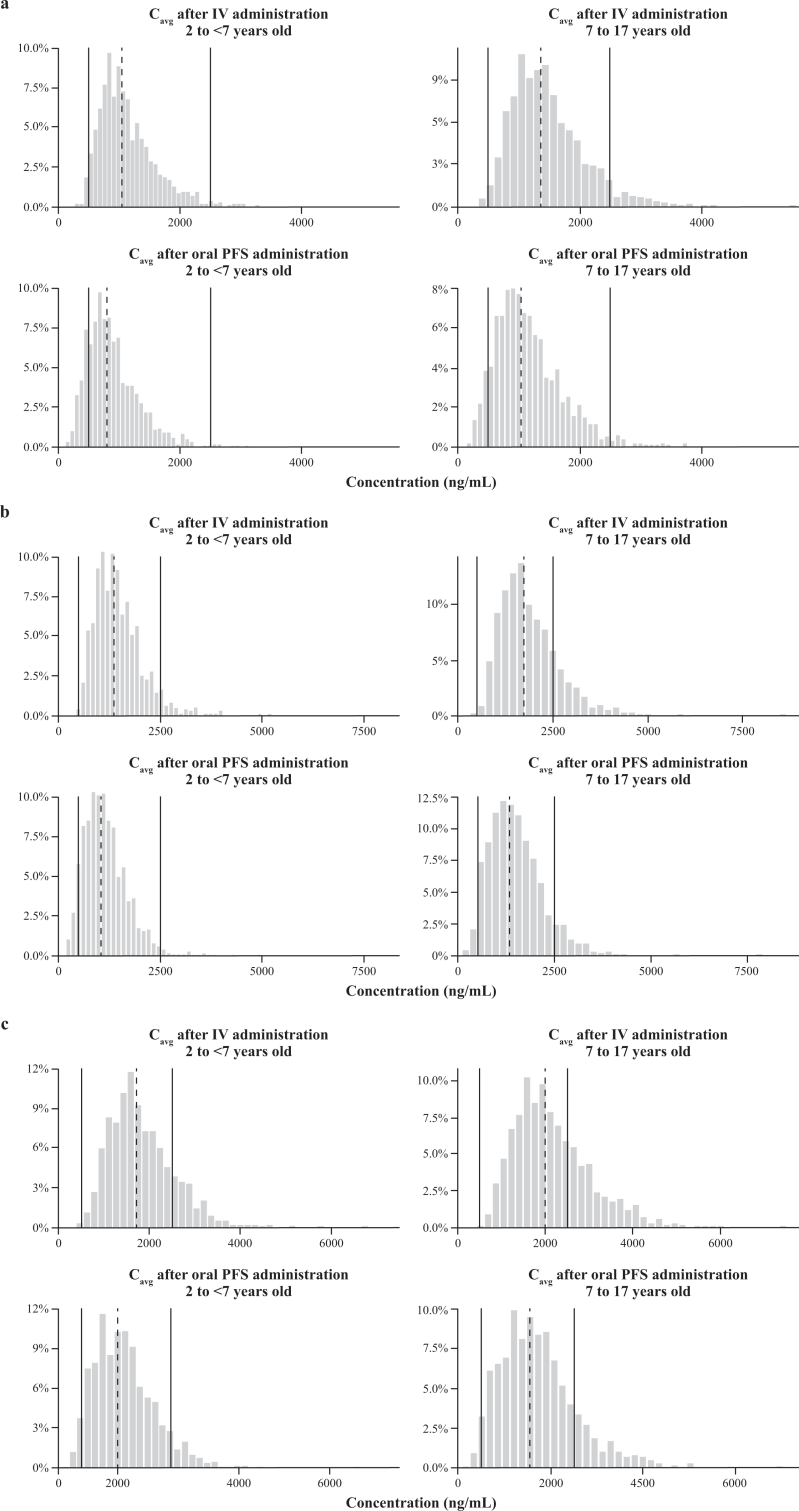
Distribution of simulated C_avg_ per age group and formulation. Distribution of simulated posaconazole C_avg_ per age group (2 to <7 or 7 to 17 years) and formulation (IV or PFS) at (a) 4.5, (b) 6, (c) and 7.5 mg/kg. The vertical dashed line is the geometric mean of the pharmacokinetic parameter. The left and right solid lines represent the thresholds of 500 and 2,500 ng/mL, respectively. C_avg_, average steady-state plasma concentration; IV, intravenous; PFS, powder for oral suspension.

The final population PK model was used to simulate PK profiles for 2,000 virtual pediatric patients (1,000 patients aged 2 to <7 years and 1,000 patients aged 7 to 17 years) for posaconazole dose regimens of 4.5, 6, or 7.5 mg/kg IV BID on day 1, followed by IV QD dosing to day 10 and oral PFS QD dosing through day 28. Both the 6- and 7.5-mg/kg dosing regimens were predicted to achieve the PK target of >500 ng/mL steady-state C_avg_ in >90% of pediatric patients in both age groups with initial IV administration for 10 days, followed by either IV or PFS administration for a further 2 wk (Fig. 3).

Overall, the simulation results for PFS and IV posaconazole administration show that the dosing regimens of both 6 and 7.5 mg/kg achieved the required steady-state C_avg_ values of >500 ng/mL in more than 90% of pediatric patients in both age group populations, irrespective of the route of administration (Table 3 ). The 7.5-mg/kg dosing regimen is predicted to achieve C_avg_ > 500 ng/mL in 4% more participants aged 2 to <7 years than the 6-mg/kg dosing regimen for PFS posaconazole; however, the 7.5-mg/kg dosing regimen in participants aged 7 to 17 years is predicted to exceed the upper C_avg_ threshold of >2,500 ng/mL in 8% (PFS and IV) and 12% (IV only) more participants than the 6 mg/kg dosing regimen. C_avg_ geometric means were predicted to be above or around the target concentration of 1,200 ng/mL at the dose regimens of 6 and 7.5 mg/kg in both age groups and for both IV and PFS administration (Table S2).

### Virtual pediatric population simulation

Simulations of a virtual pediatric population, generated from the National Health and Examination Survey (NHANES) database, were performed using the population PK model to assess the suitability of the posaconazole 300 mg tablet QD (BID on the first day) for pediatric patients >40 kg able to receive oral therapy. The distribution of body weight by age of the virtual pediatric population derived from the NHANES database is consistent with that previously observed in adults (12), with <5% of adolescents having a body weight of <40 kg.

**Fig 4:**
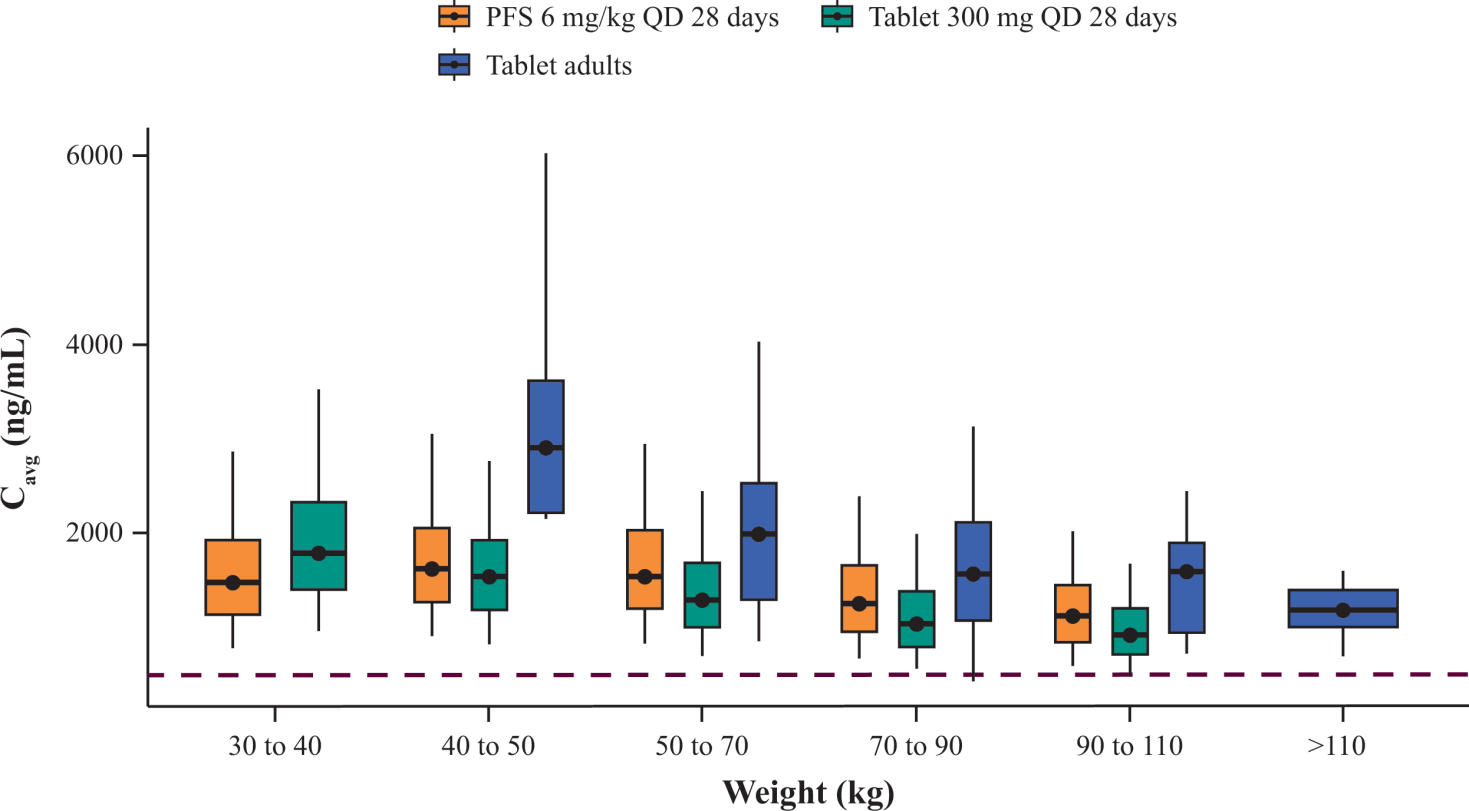
Distribution of model-predicted C_avg_ by body weight for posaconazole tablets and PFS in virtual pediatric subjects and observed distribution of C_avg_ for posaconazole tablets in adults. Frequency distribution of model-predicted C_avg_ by body weight for the 300 mg QD posaconazole tablet and the 6-mg/kg QD PFS dosing schedule in virtual pediatric patients vs the distribution of C_avg_ for the 300 mg QD posaconazole tablet observed in adults from a previous study (14), across 28 days of dosing. The line in the box represents the median, boxes extend to the 25th and 75th percentiles, and whiskers extend to the 5th and 95th percentiles. The dashed line represents the 500 ng/mL posaconazole C_avg_ target concentration. C_avg_, average steady-state plasma concentration; IV, intravenous; PFS, powder for oral suspension; QD, once daily.

For the posaconazole tablet dosing regimen of 300 mg/day, the model-predicted C_avg_ achieved the PK target of C_avg_ >500 mg/mL in 90% of pediatric patients with a body weight >40 kg (Table S3). The distribution of model-predicted C_avg_ shifted lower with increasing weight (Fig. 4). Model-predicted C_avg_ for the 300 mg tablet was 20% lower than for 300 mg PFS in patients with a body weight >50 kg, reflecting the relative bioavailability of the two formulations (PFS/tablet = 1.2).

## DISCUSSION

Plasma concentration data following IV and PFS posaconazole administration across the 3.5-, 4.5-, and 6-mg/kg per day dosing regimens in a pediatric population aged 2 to 17 years with documented or expected neutropenia at risk for IFD were well-described using a one-compartment model with first-order absorption, where bioavailability was constrained from 0 to 1 using a logit function and CL and Vc were subject to allometric scaling by body weight. Simulations predict that the 6-mg/kg per day posaconazole dose regimen will provide sufficient exposure, with >90% of participants having a C_avg_ ≥500 ng/mL following IV dosing only, or with a switch from IV to PFS administration, in both the 2- to <7-year and the 7 to 17-year age groups. Extrapolation of posaconazole PK data to a 7.5-mg/kg per day posaconazole dosing regimen was predicted to result in higher exposures in all pediatric patients; however, based on previous exposure–response analyses of pivotal posaconazole trials (11), these higher exposures are unlikely to substantially improve efficacy rates relative to the 6 mg/kg per day dose and would only modestly increase the number of patients with C_avg_ < 500 ng/mL. The predictive performance of the population PK model was confirmed by the agreement between model-predicted and observed concentration values across all the cohorts, with no obvious bias observed. Model validation approaches showed good concordance between model-predicted *post hoc* estimates of individual C_avg_ and those calculated by the noncompartmental analysis for both IV and PFS posaconazole administration as well as good concordance between model-based and observed C_min_ values. Taken together, these results support the application of the final population PK model to simulate IV and oral PFS dosing regimens in pediatric patients aged 2 to 17 years.

The 6-mg/kg per day posaconazole dosing regimen achieved the target C_avg_ geometric mean of 1,200 ng/mL for both the IV and PFS formulation in the older age group of patients aged 7 to 17 years. In the younger age group of patients aged 2 to <7 years, the target C_avg_ geometric mean was only achieved with the IV formulation. In contrast, the 4.5-mg/kg posaconazole dosing regimen did not achieve the target C_avg_ geometric mean of 1,200 ng/mL in patients aged 2 to <7 years with either the IV or PFS formulation. These results provide further support for the current US product labeling, which recommends a dosing regimen of 6 mg/kg per day (BID on the first day and up to a maximum absolute dose of 300 mg) for IV posaconazole administration in children aged 2 to 17 years. To simplify PFS administration and allow for dosing in 1-mL increments, a weight-band dosing nomogram was ultimately applied in order to maximize the proportion of pediatric patients achieving the PK target across weight bands, while also maintaining a favorable benefit–risk profile. The weight-band dosing nomogram recommended in current US product labeling is approximately 1 to 1.5 times the 6-mg/kg per day dosing regimen used in this study.

A trend toward lower exposure with PFS compared to IV administration was observed, with higher clearance in the younger age group (2 to <7 years); however, the dosing regimen of 6 mg/kg per day of either the IV or the PFS formulation still provided sufficient exposure in this age group to achieve comparable efficacy to adults. Furthermore, age was not identified as a significant covariate, supporting the conclusion that weight-based dosing at 6 mg/kg for IV administration and the use of weight-band dosing for PFS administration (up to a maximum absolute dose of 300 mg) are appropriate in the pediatric population aged 2 to 17 years, without further adjustment for age.

No significant effect on posaconazole bioavailability was identified in the population PK analysis in this study when the PFS formulation was administered with food (between 2 hours pre-dose and 1 hour post-dose). This supports current US labeling recommendations that the PFS formulation can be administered with food to pediatric patients aged 2 to 17 years (9). These findings are consistent with previously reported findings in adults, in which a high-fat meal was reported to have a minimal effect on the overall area under the curve exposure levels with the PFS formulation (data on file), although a <50% increase in the area under the curve was previously observed for a high-fat meal compared to the fasting state in adults for the 300-mg tablet formulation (15).

Results from the virtual pediatric population simulation support the use of the adult dosing regimen of a fixed-dose 300 mg/day tablet in pediatric patients with a body weight >40 kg. More than 90% of the virtual pediatric population were predicted to achieve the PK target of C_avg_ ≥500 ng/mL while remaining within the target range of exposure for which no safety issues have been identified (12, 16). As <5% of the virtual adolescent population had a body weight of <40 kg, these results are likely to be applicable to most of the adolescent population.

A limitation of this study is that a population PK model was utilized to predict posaconazole exposure levels to a 7.5-mg/kg posaconazole dosing regimen; no clinical efficacy or safety data are available for a 7.5-mg/kg posaconazole dosing regimen in pediatric patients with neutropenia or on the weight-band dosing nomogram that was ultimately approved in the United States and the European Union for PFS administration. In the P097 study, posaconazole IV and PFS formulations were well-tolerated in dosing regimens up to 6 mg/kg and had safety profiles similar to those reported for adults (14). Although safety was not assessed in the current analysis, no dose-dependent or exposure-related toxicity was identified in the pediatric population from the P097 study (14).

In conclusion, this population PK model demonstrated that a weight-based dosing regimen of IV or PFS posaconazole (up to a 300 mg absolute dose) is appropriate for the pediatric population at risk of IFD and supports current US product labeling that the PFS formulation can safely be administered with food. The 300 mg posaconazole tablet approved for use in adults was predicted to achieve the PK target and remain within a safe range of exposure in adolescent patients with a body weight of >40 kg.

## MATERIALS AND METHODS

### PK data and study population

PK data were obtained from immunocompromised patients aged 2 to 17 years, with documented or anticipated neutropenia in the setting of acute leukemia, myelodysplasia, severe aplastic anemia, autologous HSCT, high-risk neuroblastoma, advanced-stage non-Hodgkin lymphoma, allogenic HSCT during the pre-engraftment (neutropenic) period, or hemophagocytic lymphohistiocytosis, treated in the P097 study (Clinicaltrials.gov NCT02452034, Merck & Co., Inc., Rahway, NJ, USA protocol number MK-5592–097) (14). Participants received posaconazole for 10 to 28 days, initially as an IV solution, but with the option to switch to oral PFS administration between days 10 and 18 for the remainder of the treatment period. Three dose cohorts of posaconazole were assessed: 3.5, 4.5, and 6 mg/kg, administered IV BID on day 1 and IV QD on days 2 to 10, with either oral PFS or IV administration QD on days 10 to 28. The absolute maximum dose of posaconazole administered was limited to 300 mg (i.e., in patients with a high body weight in which the weight-based dosing regimen would otherwise result in an absolute dose >300 mg). The trial was conducted in accordance with the Declaration of Helsinki, Good Clinical Practice requirements, and applicable country and/or local statutes and regulations regarding Institutional Ethics Committee review, informed consent, and protection of human participants in biomedical research. All participants or their legal representatives gave written informed consent before initiation of any study procedures.

### Pharmacokinetic sampling

PK sampling during posaconazole IV administration was performed pre-dose on day 6; and pre-dose, within 15 minutes of the end of infusion; and at 4, 6, 12, and 24 hours after the start of the infusion between days 7 and 10 (14). For inpatients, PK sampling during posaconazole PFS administration was performed at pre-dose (0 hour) and 2, 4, 6, 8, and 24 hours post-dose between days 7 and 10. For outpatients, PK sampling during posaconazole PFS administration was performed at pre-dose (0 hour) and at 2 and 4 hours post-dose between days 7 and 10.

### Bioanalysis and data set preparation

Plasma posaconazole concentrations were quantified using a validated high-performance liquid chromatography with tandem mass spectrometry method (17). Measurements below the lower limit of quantification (LLOQ) of 5.00 ng/mL and missing values were excluded from the analysis, with the missing concentration-dependent variable set to 1. Pre-treatment plasma concentrations above the LLOQ were also excluded from the data set. Log-transformed plasma concentrations were used as the dependent variable. Covariates were not included in the analysis if >30% of participants did not provide a covariate value. Otherwise, the value of the missing covariate observations was imputed as the median of the remaining values from an appropriate subpopulation. For categorical covariates, such as sex and race, the most frequently occurring value was imputed. All data sets were prepared using SAS V9.3 (SAS Institute, Cary, NC).

### Population PK model development

PK data were analyzed using the NONMEM software (version 7.2; Globomax, Hanover, MD), and model fitting was performed in a UNIX environment with an Intel FORTRAN Compiler (version 11.1; Intel Corporation, Santa Clara, CA). The applied estimation method in NONMEN was the first-order conditional estimation, with an additive model for residual variability on log-transformed data. Xpose (http://xpose.sourceforge.net), perl speaks (PsN; http://psn.sourceforge.net), and R-project (version 3.4.3; http://www.r-project.org) were used for exploratory analysis and post-processing of NONMEM output (e.g., to assess goodness-of-fit).

A one-compartment model with first-order absorption was fit to the PK data set of participants administered oral PFS and IV or IV-only formulations, based on the previous analyses of posaconazole PK in adults (18). The model was parameterized in terms of CL and V_c_, with IV administration being assumed to be directed into the central compartment. Alternative structural models and refinements were explored to improve model fit, and allometric scaling of CL and V_c_ was evaluated. Residual variability in log-transformed concentrations was modeled using an additive error model, and IIV was evaluated using exponential error models. Stepwise covariate analysis assessed the influence of covariates, including age, eGFR, sex, and ethnicity, on posaconazole exposure (*p* < 0.01 for forward inclusion; *p* < 0.001 for backward deletion).

The resultant final model only contained covariates that met the pre-defined statistical criteria. As part of the final covariate selection, the clinical relevance of any relationship was also considered. The impact of food covariates on relative bioavailability was then assessed after the completion of the stepwise covariate analysis using the same statistical criteria. Information on food intake was captured within 2 hours prior to or up to 1 hour after the associated PFS dose administration and included details on whether the food intake was a light, moderate, or heavy meal. Model validation was performed using diagnostic plots, prediction-corrected visual predictive checks, and comparison of derived PK parameters (C_avg_ and C_min_) from the noncompartmental analysis of the participants treated with 3.5, 4.5, and 6 mg/kg, with those calculated from *post hoc* estimates.

### Final population PK model simulations

The final population PK model was used to simulate the distribution of posaconazole exposure in both the 2- to <7-year age group and the 7- to 17-year age group. The model was used to derive the proportion of pediatric patients achieving an exposure range of C_avg_ ≥500 ng/mL (the primary concentration target) as well as the proportion of pediatric patients achieving an exposure range of C_min_ ≥500 ng/mL (a secondary concentration target). The exposure target was a mean C_avg_ of 1,200 ng/mL, with approximately 90% of participants with C_avg_ ≥500 to <2,500 ng/mL; the critical target in this study was achieving C_avg_ ≥ 500 ng/mL in 90% of participants. The dose levels included in the simulations were 4.5, 6, or 7.5 mg/kg, with the maximum absolute dose limited to 300 mg. The 7.5-mg/kg regimen, a higher dose than was evaluated in the clinical study, was included for the purpose of confirming dose recommendations. Steady-state exposure parameters (C_avg_; C_min_) were calculated after the last dose of IV and PFS administration.

Simulations were performed using a combination of R and NONMEM. R was used to create the data sets (1,000 subjects aged 2 to <7 years and 1,000 subjects aged 7 to 17 years) and jointly sample the age and weight from a combined pediatric population from the P097 (14) and P032 studies. The addition of data from P032 was anticipated to enable a more robust assessment of the distribution of posaconazole exposures across a relevant pediatric population, as both studies included pediatric patients with similar diagnoses and demographics.

### Virtual pediatric population analysis

As there were insufficient data in the weight range of interest in the P097 study to allow a resampling approach, a virtual pediatric population was generated using the NHANES database. The distribution of body weight by age of the virtual pediatric population derived from the NHANES database (19) was consistent with the body weight by age distribution observed in the P097 and P032 studies. The virtual pediatric population was used to predict the population PK for the posaconazole tablet formulation in pediatric patients with a body weight >40 kg.

## Data Availability

The data sharing policy, including restrictions, of Merck Sharp & Dohme LLC, a subsidiary of Merck & Co., Inc., Rahway, NJ, USA, is available at http://engagezone.msd.com/ds_documentation.php. Requests for access to the clinical study data can be submitted through the Engage Zone site or via email to the Data Access mailbox.

## SUPPLEMENTAL MATERIAL

Supplemental tables and figures aac.01197-23-s0001.pdf Tables S1-S3 and Fig. S1-S4.

ASM does not own the copyrights to Supplemental Material that may be linked to, or accessed through, an article. The authors have granted ASM a non-exclusive, world-wide license to publish the Supplemental Material files. Please contact the corresponding author directly for reuse.
