# Supplementary material for: A population pharmacokinetic model for posaconazole intravenous solution and oral powder for suspension formulations in pediatric patients with neutropenia
Source: Antimicrob Agents Chemother. 2024 Feb 20;68(4):e01197-23. doi: 10.1128/aac.01197-23 (PMC10994819; doi:10.1128/aac.01197-23)

SUPPLEMENT

A Population Pharmacokinetic Model for Posaconazole Intravenous Solution and Oral Powder for Suspension Formulations in Pediatric Patients With Neutropenia

Authors: Gregory Winchell,[a] Rik de Greef,[a] Aziz Ouerdani,[a] Floris Fauchet,[a] Rebecca E. Wrishko,[b] Eric Mangin,[b] Christopher Bruno,[b] and Hetty Waskin[b]

Affiliations:

[a]Certara Netherlands B.V., Oss, Netherlands

[b]Merck & Co., Inc., Rahway, New Jersey, USA

Supplementary TABLE 1 Number of patients by age group, dose, and route of posaconazole administration

Age group	Dose	Route of administration	Number of patients
2 to <7 years	3.5 mg/kg	IV and PFS	11
		IV only	3
	4.5 mg/kg	IV and PFS	6
		IV only	9
	6 mg/kg	IV and PFS	7
		IV only	12
7 to 17 years	3.5 mg/kg	IV and PFS	12
		IV only	9
	4.5 mg/kg	IV and PFS	8
		IV only	8
	6 mg/kg	IV and PFS	16
		IV only	13

IV, intravenous; PFS, power for oral suspension.

Supplementary TABLE 2 Predicted geometric mean C_{avg} and C_{min} across dose regimens by age group

Parameter	Formulation	2 to <7 years of age		7 to 17 years of age	
		Geometric mean, ng/mL	Geometric CV, %	Geometric mean, ng/mL	Geometric CV, %
		4.5 mg/kg		4.5 mg/kg	
C_{avg}	IV	1044.94	40.34	1361.00	41.98
	PFS	796.61	50.53	1041.55	51.96
C_{min}	IV	515.44	70.17	795.48	64.06
	PFS	504.96	65.77	734.80	63.39
		6 mg/kg		6 mg/kg	
C_{avg}	IV	1364.60	39.18	1748.46	40.66
	PFS	1049.68	50.36	1331.39	51.33
C_{min}	IV	656.24	69.65	1035.21	61.37
	PFS	654.95	65.66	947.19	62.21
		7.5 mg/kg		7.5 mg/kg	
C_{avg}	IV	1713.79	39.65	2001.55	41.54
	PFS	1320.77	50.85	1546.59	53.08
C_{min}	IV	830.09	70.76	1210.21	57.99
	PFS	827.78	67.18	1114.93	61.66

C_{avg}, average plasma concentration during dosing interval; C_{min}, trough plasma concentration; CV, coefficient of variation; IV, intravenous; PFS, powder for oral suspension.

Supplementary TABLE 3 Percentage of subjects with C_{avg} below, within, and above the pre-specified posaconazole C_{avg} target range by weight for posaconazole tablets and PFS in virtual pediatric patients versus tablets in adults

Weight categories	Percentage of patients with C_{avg}								
	300-mg tablets (pediatric)			PFS 6 mg/kg (pediatric)			300-mg tablets (adults)		
	<500 ng/mL	≥500 to <2500 ng/mL	≥2500 ng/mL	<500 ng/mL	≥500 to <2500 ng/mL	≥2500 ng/mL	<500 ng/mL	≥500 to <2500 ng/mL	≥2500 ng/mL
30-40 kg	0.10%	80.10%	19.80%	0.30%	89%	10.70%	--	--	--
40-50 kg	0%	90.90%	9.10%	0%	87.80%	12.20%	0%	50%	50%
50-70 kg	0.60%	95.30%	4.10%	0.10%	88.10%	11.80%	1.33%	72%	26.67%
70-90 kg	2.90%	96%	1.10%	0.50%	95.80%	3.70%	7.62%	80.95%	11.43%
90-110 kg	5%	94.60%	0.40%	1.40%	96.60%	2%	0%	96.88%	3.12%
>110 kg	Not determined						0%	100%	0%

C_{avg}, average plasma concentration during dosing interval; PFS, powder for oral suspension.

Supplementary FIG 1

ETA shrinkages presented as distributions of the individual estimates for the random effects of (A) clearance, (B) volume of distribution, and (C) relative bioavailability. The variability for the relative bioavailability was implemented to follow a normal distribution in the logit domain. Dashed black line represents the observed ETA distribution. Solid black line represents the expected ETA distribution. CL, clearance; F1, relative bioavailability; V_c, central volume of distribution.

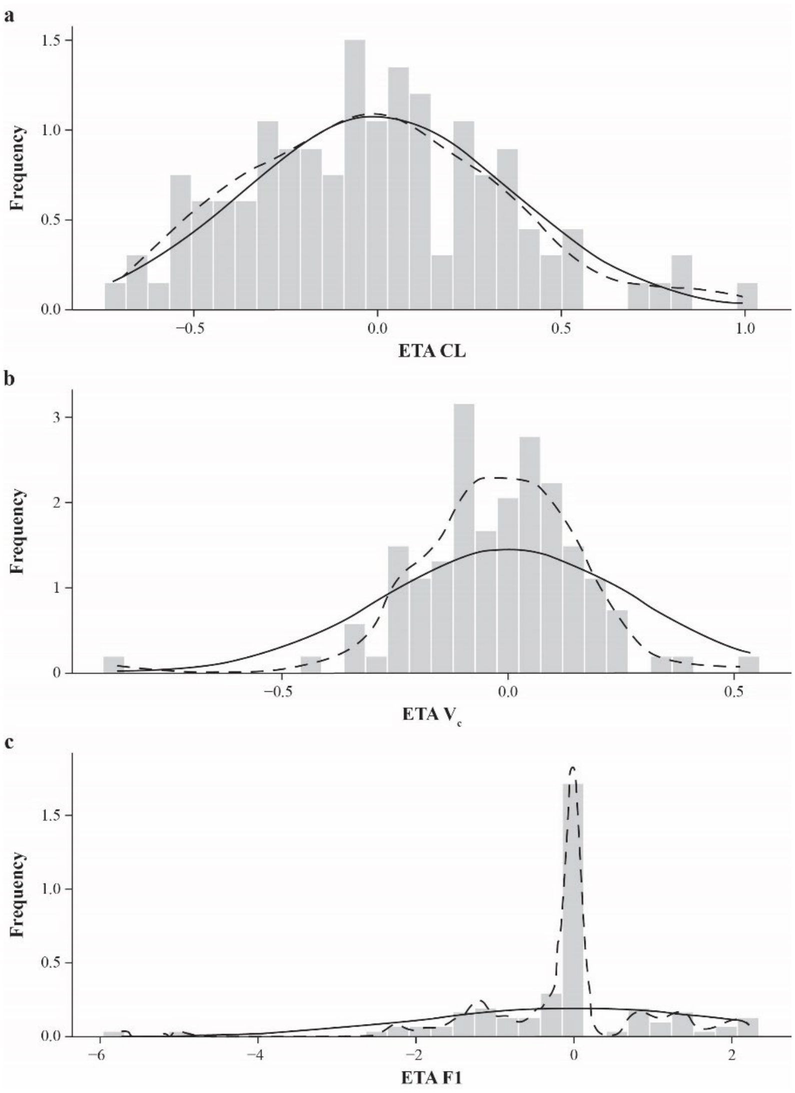

Supplementary FIG 2

Effects of (A) age and (B) weight on interindividual variability for CL, V_c, and F1. Dots represent individual data. Solid grey lines are trend lines. CL, clearance; F1, relative bioavailability; IIV, interindividual variability; V_c, central volume of distribution.

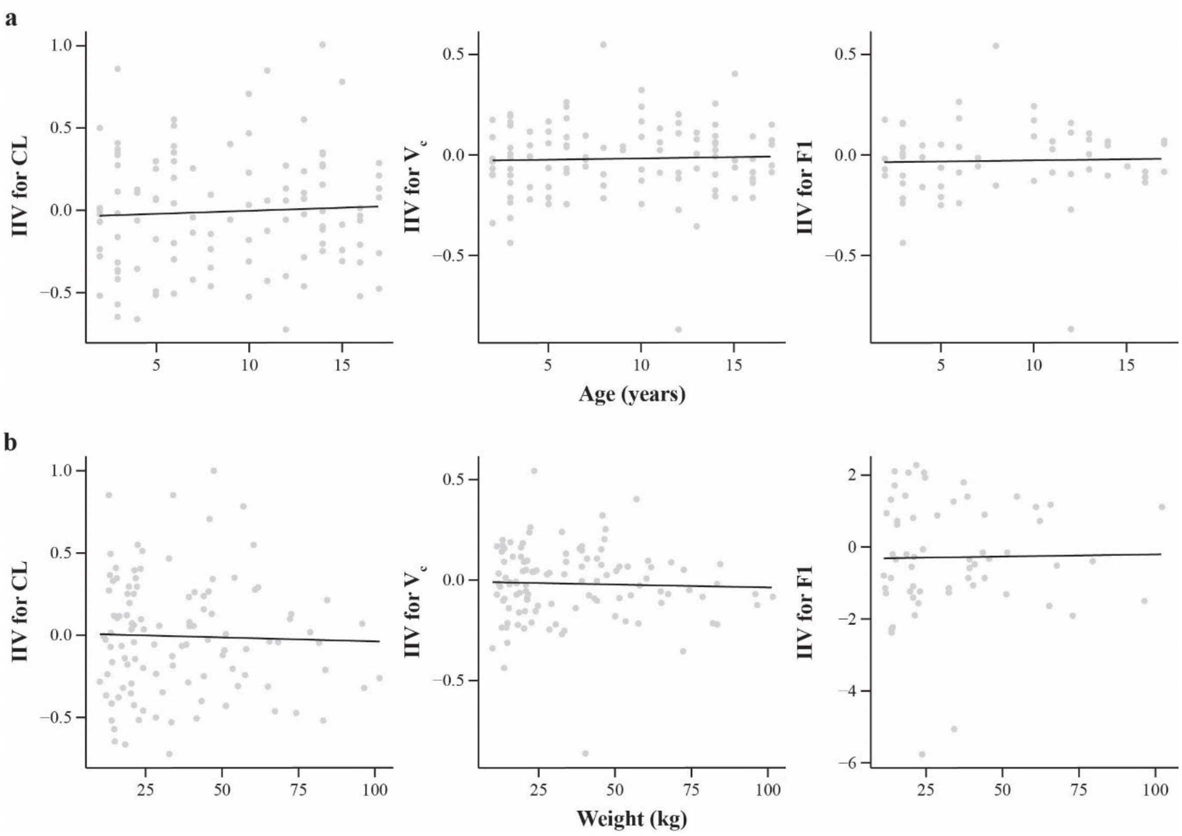

Supplementary FIG 3

Pharmacokinetic parameters C_{avg} (upper panels) and C_{min} (lower panels) derived from the model-based post hoc parameter estimates versus parameter estimates obtained from the noncompartmental analysis for both IV and oral PFS administration for dosing regimens of 3.5, 4.5, and 6 mg/kg. The black dotted line represents the identity line. The blue dotted line represents the Loess line for the 6 mg/kg dosing regimen. The green dotted line represents the Loess line for the 4.5 mg/kg dosing regimen. The red dotted line represents the Loess line for the 3.5 mg/kg dosing regimen. C_{avg}, average steady-state concentration; C_{min}, trough plasma concentration; IV, intravenous; NCA, noncompartmental analysis; PFS, powder for oral suspension; PK, pharmacokinetic; QD, once daily.

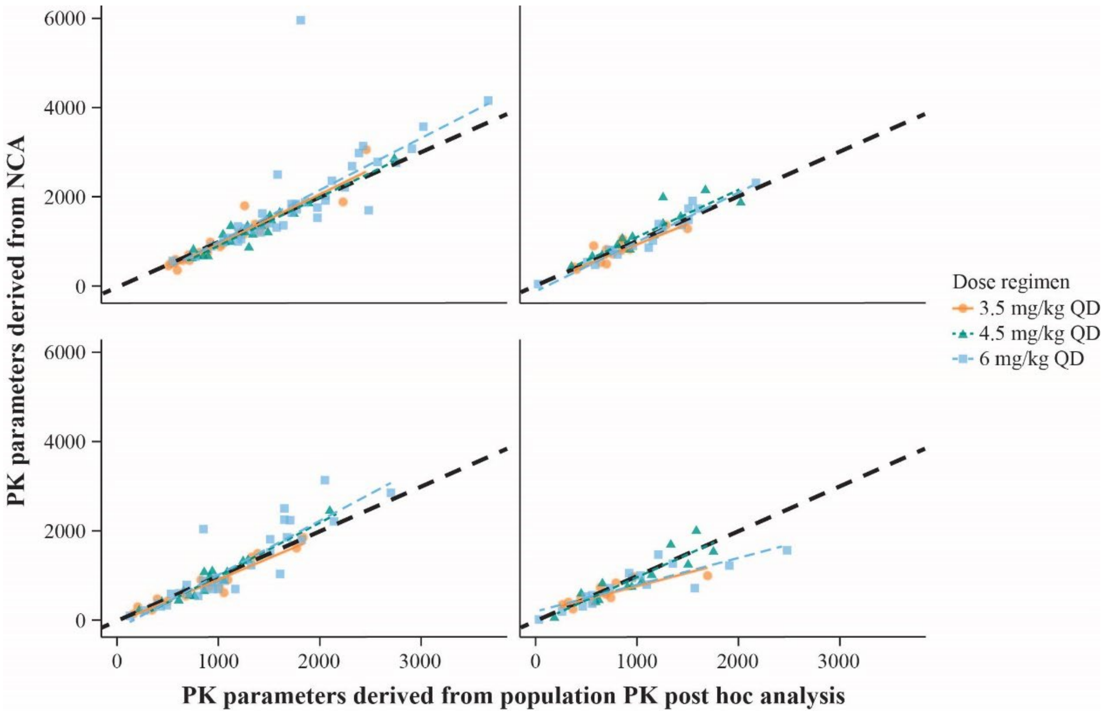

Supplementary FIG 4

Prediction-corrected visual predictive checks of log-scale posaconazole plasma concentration versus time, stratified by age group and route of administration. Datasets were simulated based on the fixed and random effect estimates of the final model. Visual predictive checks were performed on 500 replicate simulations. The 5th, 50th and 95th percentiles were calculated from the simulated profiles and were superimposed on the raw data to allow assessment of model predictability. IV, intravenous.

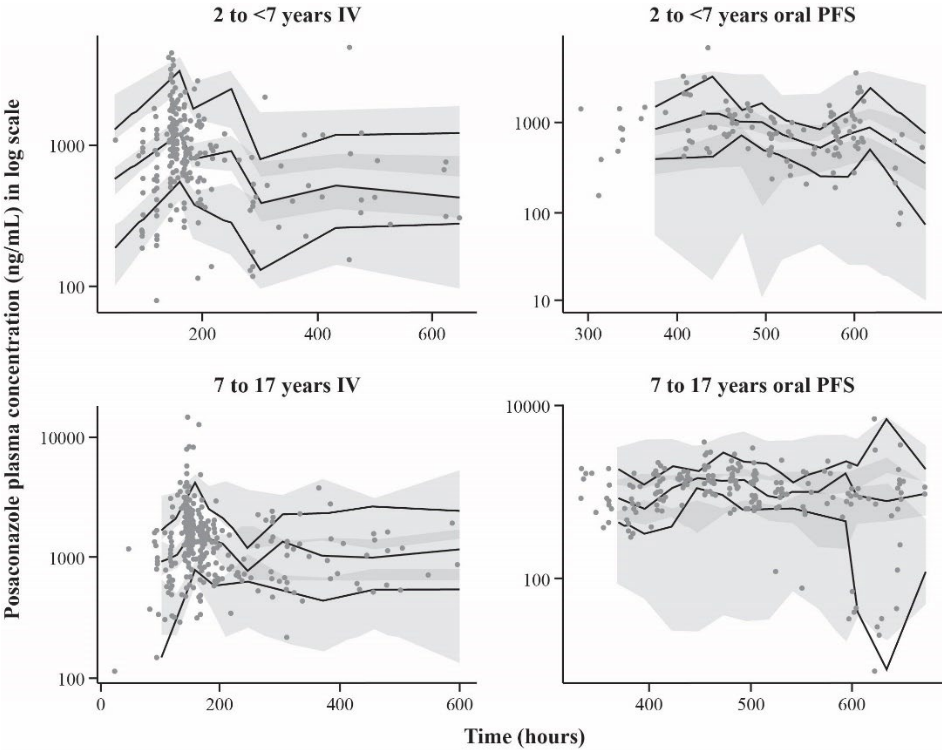

Supplement: Supplemental tables and figures — Tables S1-S3 and Fig. S1-S4. [file aac.01197-23-s0001.pdf]
